# Association of a Simplified Finnegan Neonatal Abstinence Scoring Tool With the Need for Pharmacologic Treatment for Neonatal Abstinence Syndrome

**DOI:** 10.1001/jamanetworkopen.2020.2275

**Published:** 2020-04-08

**Authors:** Lori A. Devlin, Janis L. Breeze, Norma Terrin, Enrique Gomez Pomar, Henrietta Bada, Loretta P. Finnegan, Kevin E. O’Grady, Hendrée E. Jones, Barry Lester, Jonathan M. Davis

**Affiliations:** 1 Department of Pediatrics, University of Louisville School of Medicine, Louisville, Kentucky; 2 Tufts Clinical and Translational Science Institute, Boston, Massachusetts; 3 Institute for Clinical Research and Health Policy Studies, Tufts Medical Center, Boston, Massachusetts; 4 University of Kentucky School of Medicine, Lexington; 5 The College on Problems of Drug Dependence, Philadelphia, Pennsylvania; 6 University of Maryland, College Park; 7 University of North Carolina, Chapel Hill; 8 Women and Infants Hospital, Providence, Rhode Island; 9 The Floating Hospital for Children at Tufts Medical Center, Boston, Massachusetts

## Abstract

**Importance:**

Observer-rated scales, such as the Finnegan Neonatal Abstinence Scoring Tool (FNAST), are used to quantify the severity of neonatal abstinence syndrome (NAS) and guide pharmacologic therapy. The FNAST, a comprehensive 21-item assessment tool, was developed for research and subsequently integrated into clinical practice; a simpler tool, designed to account for clinically meaningful outcomes, is urgently needed to standardize assessment.

**Objectives:**

To identify FNAST items independently associated with the decision to use pharmacologic therapy and to simplify the FNAST while minimizing loss of information for the treatment decision.

**Design, Setting, and Participants:**

This multisite cohort study included 424 neonates with opioid exposure who had a gestational age of at least 36 weeks with follow-up from birth to hospital discharge in the derivation cohort and 109 neonates with opioid exposure from the Maternal Opioid Treatment: Human Experimental Research Study in the validation cohort. Neonates in the derivation cohort were included in a medical record review at the Universities of Louisville and Kentucky or in a randomized clinical trial and observational study conducted at Tufts University (2014-2018); the Maternal Opioid Treatment: Human Experimental Research was conducted from 2005 to 2008. Data analysis was conducted from May 2017 to August 2019.

**Exposures:**

Prenatal opioid exposure.

**Main Outcomes and Measures:**

All FNAST items were dichotomized as present or not present, and logistic regression was used to identify binary items independently associated with pharmacologic treatment. The final model was validated with an independent cohort of neonates with opioid exposure.

**Results:**

Among 424 neonates (gestational age, ≥36 weeks; 217 [51%] female infants), convulsions were not observed, and high-pitched cry and hyperactive Moro reflex had extremely different frequencies across cohorts. Therefore, these 3 FNAST items were removed from further analysis. The 2 tremor items were combined, and 8 of the remaining 17 items were independently associated with pharmacologic treatment, with an area under the curve of 0.86 (95% CI, 0.82-0.89) compared with 0.90 (95% CI, 0.87-0.94) for the 21-item FNAST. External validation of the 8 items resulted in an area under the curve of 0.86 (95% CI, 0.79-0.93). Thresholds of 4 and 5 on the simplified scale yielded the closest agreement with FNAST thresholds of 8 and 12 (weighted κ = 0.55; 95% CI, 0.48-0.61).

**Conclusions and Relevance:**

The findings of this study suggest that 8 signs of NAS may be sufficient to assess whether a neonate meets criteria for pharmacologic therapy. A focus on these signs could simplify the FNAST tool and may enhance its clinical utility.

## Key Points

**Question:**

Can a simplified Finnegan Neonatal Abstinence Scoring Tool (FNAST), composed only of dichotomized items that are independently associated with the decision to initiate pharmacologic therapy, discriminate between infants who did and did not receive therapy as effectively as the original FNAST?

**Findings:**

In this cohort study of 424 neonates with opioid exposure, the simplified FNAST discriminated between neonates who did and did not receive pharmacologic treatment nearly as well as the original FNAST.

**Meaning:**

Use of a simplified FNAST may provide an accurate means of identifying neonatal abstinence syndrome and may enhance the clinical utility of the tool.

## Teaser

This cohort study identifies items on the Finnegan Neonatal Abstinence Scoring Tool (FNAST) that are independently associated with the decision to use pharmacologic therapy and simplifies the tool while minimizing loss of information for the treatment decision.

## Introduction

The medical and nonmedical use of opioids in the United States has increased significantly during the last decade.^1,2,3,4^ Opioid use disorder (OUD) during pregnancy increased from 1.5 per 1000 live births to 6.5 per 1000 live births between 1999 and 2014.^5^ Antenatal exposure to opioids has led to a 5-fold rise in the incidence of neonatal abstinence syndrome (NAS) between 2004 and 2014, a number that continues to increase.^6,7^ Neonatal abstinence syndrome is a withdrawal syndrome that occurs after the interruption of the passive transfer of maternal opioids at the time of birth. The diagnosis of NAS is dependent on the presence of key signs of withdrawal, with severity being highly variable.^8,9^ Every neonate exposed to opioids in utero is somewhere along the continuum of withdrawal. While some neonates have mild signs and normal physiologic functions, others have more severe NAS that requires pharmacologic treatment to avoid major complications.^10^ Differences in the expression of NAS is associated with many factors, including the type(s) of opioid exposure, coexposure with other illicit drugs and/or psychotropic medications, genetic and epigenetic variability, the gestational age and sex of the neonate, breastfeeding, and parental engagement.^11,12,13,14,15,16,17^

Observer-rated scales have been used for more than 40 years to assess the severity of NAS and guide the initiation and adjustment of pharmacologic therapy.^18,19,20,21^ Differences between raters in the assessment of NAS have been associated with significant differences in initiation and duration of pharmacologic therapy, length of hospital stay, and health care utilization.^22^ The Finnegan Neonatal Abstinence Scoring Tool (FNAST) is commonly used for the assessment of neonates with NAS.^23,24,25^ It is a screening tool that comprises 21 items, with many items having 2 to 4 subcategories and weighting for each category, which varies from 1 to 5.^18,19^ Although differences among raters have been observed with the use of the FNAST,^26,27^ these rater differences can be minimized with a comprehensive educational approach that optimizes and then maintains interobserver reliability.^22,26,27,28,29^ Several tools have been developed to shorten and simplify the FNAST,^27,30,31,32,33^ but existing studies are limited by small cohorts that lack generalizability, study samples that do not differentiate between neonates with and without pharmacologic treatment, and a lack of external validation of the findings.

The goal of this study was to significantly shorten and simplify the original FNAST by focusing on signs of withdrawal that prompt clinical intervention. In this article, we address the following questions. First, is information lost by dichotomizing components of the FNAST and eliminating weighting? Second, are there large differences among cohorts in how frequently specific signs are observed? Third, which binary-coded FNAST items are independently associated with the receipt of pharmacologic therapy?

## Methods

Retrospective medical record reviews of consecutive neonates with antenatal opioid exposure were conducted at the University of Louisville and University of Kentucky for infants born in 2014. In addition, prospective data were obtained from an 8-site clinical trial that compared methadone with morphine for the treatment of NAS (conducted 2014-2018, led by Tufts University)^34^ and from a concurrent observational study of neonates whose parents gave consent for the clinical trial but did not require treatment or whose parents refused consent for randomization in the clinical trial but consented to data collection (eFigure in the Supplement). Included neonates had a gestational age of at least 36 weeks and had no other significant medical or surgical illness. All sites used the FNAST for assessment, and nonpharmacologic care was the initial treatment for NAS in this study population.

Data from an external cohort of neonates enrolled in the Maternal Opioid Treatment: Human Experimental Research (MOTHER) study (conducted 2005-2008) were used to validate the final model.^26^ Of the 131 infants in the original study, 109 met inclusion criteria for this analysis (ie, gestational age ≥36 weeks; adequate data on timing of treatment initiation; complete FNAST data on day of treatment or on day 3 of life if not treated). The MOTHER NAS (MNS) tool included 28 items (the FNAST plus 7 additional items). Therefore, data for every FNAST item were available at each assessment in the MOTHER data set. The scores used to initiate pharmacologic therapy in the MOTHER study were calculated from 19 of the 28 MNS items, including 3 that are not on the FNAST.

Each site’s institutional review board approved the study. The deidentified retrospective medical record review from the Kentucky sites was conducted under a waiver of informed consent, and informed consent was obtained for patients included in the clinical trial, observational cohort, and the MOTHER study. This study followed the Strengthening the Reporting of Observational Studies in Epidemiology (STROBE) reporting guideline. Analyses were conducted from May 2017 to August 2019.

The present analysis used a single assessment point for each neonate, ie, the time of the highest score on the day the neonate initially received pharmacologic therapy for NAS or on day 3 of life if never treated. The third day was selected for analyzing untreated neonates because it was the median day when treatment was initiated among neonates who were treated. The criteria for initial treatment for NAS varied by site and included: 3 consecutive scores of at least 8 or 2 consecutive scores of at least 12 on the FNAST at the Kentucky sites; 2 consecutive scores of at least 8 or 1 score of at least 12 on the FNAST for the multisite study; and 2 consecutive scores of at least 9 or 1 score of at least 13 on the NAS scale for the MOTHER study.

Items on the FNAST with multiple categories were dichotomized as present or absent; if any sign was recorded, the neonate was coded as having the sign (eg, hyperactive Moro reflex and markedly hyperactive Moro reflex were both coded as hyperactive Moro reflex). The 2 items related to tremors were combined to form a single binary item.

### Statistical Analysis

Differences among the 3 cohorts in the frequency of each binary item were tested using a χ^2^ test for proportions. Endorsement of each item across cohorts was also compared using the area under the curve (AUC). Items that differed by more than 50 percentage points among cohorts and items that were never observed were excluded from subsequent analyses. Forward stepwise multivariable logistic regression was used to determine which of the remaining items were independently associated with receipt of pharmacologic therapy, adjusting for cohort. The criteria for stepping items into the model and retaining items in the model were *P* < .10 and *P* < .05, respectively. Model discrimination was evaluated using the AUC. The selected items were validated by calculating the AUC of a multivariable logistic regression on the validation data set. Thresholds for the new score were selected to optimize agreement between it and the FNAST in categorizing scores into 3 groups: low (<8 on FNAST), medium (≥8 to <12), and high (≥12).

Statistical analysis was conducted with SAS statistical software version 9.4 (SAS Institute). Except as stated for model building, statistical significance was set at *P* < .05, and all tests were 2-tailed.

## Results

A total of 424 neonates were included in the primary analysis (mean [SD] birth weight, 3122 [618] g; 217 [51%] female infants). Among 238 treated neonates, the median (interquartile range) time to treatment was 3 (2-4) days from birth. The frequency of each item in the FNAST is shown in Table 1. Generalized convulsions, fever (body temperature ≥38.4 °C), and vomiting (projectile) were not reported. Many of the items had statistically significant differences in percentage endorsement across cohorts (eg, sleeps <3 h: Louisville group, 70 [55.1%]; Tufts group, 108 [53.2%]; Kentucky group, 80 [85.1%]; *P* < .001; body temperature ≥37.2 °C: Louisville group, 30 [23.6%]; Tufts group, 81 [39.9%]; Kentucky group, 18 [19.1%]; *P* < .001), but such differences only appeared extreme (ie, greater than 50 percentage points) in the case of high-pitched crying (Louisville group, 98 [77.2%]; Tufts group, 42 [20.7%]; Kentucky group, 75 [79.8%]; *P* < .001) and hyperactive Moro reflex (Louisville group, 36 [28.3%]; Tufts group, 35 [17.2%]; Kentucky group, 64 [68.1%]; *P* < .001) (Table 2). These 2 items also had the highest AUCs in models comparing endorsement of the item among cohorts (crying: 0.79 [95% CI, 0.75-0.83]; Moro reflex: 0.72 [95% CI, 0.62-0.77]). Thus, these 2 items as well as generalized convulsions (which were not observed) were excluded from subsequent model building.

**Table 1.** Original 21-Item Finnegan Neonatal Abstinence Scoring Tool Frequencies Among 424 Neonates

Original item	Score	No. (%)
High-pitched crying		
Excessive	2	187 (44.1)
Continuous	3	28 (6.6)
Sleeps after feeding, h		
<3	1	83 (19.6)
<2	2	96 (22.6)
<1	3	79 (18.6)
Moro reflex		
Hyperactive	2	127 (30.0)
Markedly hyperactive	3	8 (1.9)
Tremors when disturbed		
Mild	1	201 (47.4)
Moderate to severe	2	145 (34.2)
Tremors when undisturbed		
Mild	3	88 (20.8)
Moderate to severe	4	58 (13.7)
Increased muscle tone	2	389 (91.8)
Excoriation	1	104 (24.5)
Myoclonic jerks	3	13 (3.1)
Generalized convulsions	5	0
Sweating	1	26 (6.1)
Body temperature, °C		
37.2-38.3	1	129 (30.4)
≥38.4	2	0
Yawning >3 times/scoring interval	1	21 (5.0)
Mottling	1	124 (29.2)
Nasal stuffiness	1	59 (13.9)
Sneezing >3 times/scoring interval	1	148 (34.9)
Nasal flaring	2	17 (4.0)
Respiratory rate		
>60/min	1	138 (32.6)
>60/min with retractions	2	20 (4.7)
Excessive sucking	1	196 (46.2)
Poor feeding	2	100 (23.6)
Regurgitation	2	76 (17.9)
Projectile vomiting	3	0
Stools		
Loose	2	88 (20.8)
Watery	3	16 (3.8)

**Table 2.** Differences in Percentage Endorsement of Finnegan Neonatal Abstinence Scoring Tool Items Among 424 Neonates

Binary Item	No. (%)			*P* value	MOTHER (n = 109), No. (%)
	Louisville (n = 127)	Tufts (n = 203)	University of Kentucky (n = 94)		
High-pitched crying	98 (77.2)	42 (20.7)	75 (79.8)	<.001	31 (28.4)
Sleeps <3 h after feeding	70 (55.1)	108 (53.2)	80 (85.1)	<.001	82 (75.2)
Hyperactive Moro reflex	36 (28.3)	35 (17.2)	64 (68.1)	<.001	42 (38.5)
Tremors when disturbed	119 (93.7)	163 (80.3)	64 (68.1)	<.001	85 (78.0)
Tremors when undisturbed	60 (47.2)	67 (33.0)	19 (20.2)	<.001	41 (37.6)
Increased muscle tone	120 (94.5)	182 (89.7)	87 (92.6)	.28	99 (90.8)
Excoriation	36 (28.3)	48 (23.6)	20 (21.3)	.44	21 (19.3)
Myoclonic jerks	5 (3.9)	3 (1.5)	5 (5.3)	.16	1 (0.9)
Sweating	8 (6.3)	10 (4.9)	8 (8.5)	.49	6 (5.5)
Body temperature ≥37.2 °C	30 (23.6)	81 (39.9)	18 (19.1)	<.001	1 (0.9)
Yawning >3 times/scoring interval	4 (3.1)	14 (6.9)	3 (3.2)	.21	4 (3.7)
Mottling	55 (43.3)	50 (24.6)	19 (20.2)	<.001	13 (11.9)
Nasal stuffiness	25 (19.7)	21 (10.3)	13 (13.8)	.06	13 (11.9)
Sneezing >3 times/scoring interval	40 (31.5)	67 (33.0)	41 (43.6)	.13	34 (31.2)
Nasal flaring	4 (3.1)	5 (2.5)	8 (8.5)	.04	3 (2.8)
Respiratory rate >60/min	57 (44.9)	60 (29.6)	41 (43.6)	.007	53 (48.6)
Excessive sucking	72 (56.7)	63 (31.0)	61 (64.9)	<.001	29 (26.6)
Poor feeding	33 (26.0)	35 (17.2)	32 (34.0)	.005	31 (28.4)
Regurgitation	25 (19.7)	24 (11.8)	27 (28.7)	.002	19 (17.4)
Loose or watery stools	32 (25.2)	34 (16.7)	38 (40.4)	<.001	28 (25.7)

Abbreviation: MOTHER, Maternal Opioid Treatment: Human Experimental Research.

The stepwise regression selected 8 items that were independently associated with receipt of pharmacologic therapy (sleeps <3 hours after feeding: odds ratio [OR], 3.1; 95% CI, 1.9-5.0; *P* < .001; tremors when disturbed: OR, 2.3; 95% CI, 1.3-3.9; *P* = .003; tremors when undisturbed: OR, 3.5; 95% CI, 2.0-6.2; *P* < .001; increased muscle tone: OR, 10.0; 95% CI, 3.9-26.0; *P* < .001; body temperature ≥37.2 °C: OR, 2.1; 95% CI, 1.3-3.5; *P* = .002; respiratory rate >60/min: OR, 2.6; 95% CI, 1.6-4.1; *P* < .001; excessive sucking: OR, 2.4; 95% CI, 1.5-3.8; *P* < .001; poor feeding: OR, 3.0; 95% CI, 1.7-5.3; *P* < .001; regurgitation: OR, 3.2; 95% CI, 1.7-6.2; *P* < .001) (Table 3). The AUC for the 8-item model was 0.86 (95% CI, 0.82-0.89) compared with 0.90 (95% CI, 0.87-0.94) for the 21-item FNAST. When validated with the cohort from the MOTHER study, the model had an AUC of 0.86 (95% CI, 0.79-0.93). Comparison of the original and simplified scales is seen in Table 4. For example, the 3 levels of sleeps after feeding were collapsed into 1 category; similarly, the 2 categories of fever were collapsed into 1. Thresholds of 4 and 5 on the simplified scale yielded the closest agreement with FNAST thresholds of 8 and 12, with a weighted κ of 0.55 (95% CI, 0.48-0.61) (Table 5).

**Table 3.** Logistic Regression Analyses Predicting Pharmacotherapy From the Original and Simplified Finnegan Neonatal Abstinence Scoring Tool Items Among 424 Neonates

Original binary item	Univariate analysis^a^		Simplified model with 8 items ^a^^,^^b^	
	OR (95% CI)	*P* value	OR (95% CI)	*P* value
High-pitched crying	4.1 (2.0-8.1)	<.001	NA	NA
Sleeps <3 h after feeding	3.1 (1.9-5.0)	<.001	2.8 (1.6-4.8)	<.001
Hyperactive Moro reflex	3.5 (2.0-6.2)	<.001	NA	NA
Tremors				
When disturbed	2.3 (1.3-3.9)	.003	4.6 (2.0-10.2)	<.001
When undisturbed	5.0 (2.9-8.7)	<.001		
Increased muscle tone	10.0 (3.9-26.0)	<.001	9.1 (3.0-27.8)	<.001
Excoriation	2.1 (1.2-3.5)	.007	NA	NA
Myoclonic jerks	7.1 (0.9-57.4)	.07	NA	NA
Generalized convulsions	NA	NA	NA	NA
Sweating	5.2 (1.5-18.1)	.01	NA	NA
Body temperature ≥37.2 °C	2.1 (1.3-3.5)	.002	1.9 (1.1-3.3)	.0318
Yawning >3 times/scoring interval	1.4 (0.5-3.7)	.48	NA	NA
Mottling	1.4 (0.8-2.3)	.19	NA	NA
Nasal stuffiness	1.5 (0.8-2.9)	.25	NA	NA
Sneezing >3 times/scoring interval	1.8 (1.1-2.8)	.01	NA	NA
Nasal flaring	2.2 (0.7-7.2)	.19	NA	NA
Respiratory rate >60/min	2.6 (1.6-4.1)	<.001	2.0 (1.1-3.4)	.01
Excessive sucking	2.4 (1.5-3.8)	<.001	2.0 (1.2-3.5)	.01
Poor feeding	3.0 (1.7-5.3)	<.001	2.7 (1.4-5.3)	.004
Regurgitation	3.2 (1.7-6.2)	<.001	4.0 (1.8-8.8)	<.001
Loose or watery stools	1.5 (0.9-2.5)	.13	NA	NA

Abbreviations: NA, not applicable; OR, odds ratio.

^a^ All models adjusted for cohort.

^b^ Area under the curve for simplified model was 0.86 (95% CI, 0.82-0.89). Cells marked with NA indicate that the original binary item was not retained in the simplified Finnegan Neonatal Abstinence Scoring Tool.

**Table 4.** Original 21-Item and Simplified 8-Item FNAST Scoring

FNAST		Simplified FNAST^a^	
Original item	Score	New item	Score
High-pitched crying		NA	NA
Excessive	2	NA	NA
Continuous	3		
Sleeps after feeding, h			
<1	3	Sleeps <3 h after feeding	1
<2	2		
<3	1		
Moro reflex			
Hyperactive	2	NA	NA
Markedly hyperactive	3		
Tremors when disturbed			
Mild	1	Any tremors	1
Moderate to severe	2		
Tremors when undisturbed			
Mild	3	NA	NA
Moderate to severe	4		
Increased muscle tone	2	Increased muscle tone	1
Excoriation	1	NA	NA
Myoclonic jerks	3	NA	NA
Generalized convulsions	5	NA	NA
Sweating	1	NA	NA
Body temperature, °C			
37.2-38.3	1	Body temperature ≥37.2 °C	1
≥38.4	2		
Yawning >3 times/scoring interval	1	NA	NA
Mottling	1	NA	NA
Nasal stuffiness	1	NA	NA
Sneezing >3 times/scoring interval	1	NA	NA
Nasal flaring	2	NA	NA
Respiratory rate			
>60/min	1	Respiratory rate >60 min	1
>60/min with retractions	2		
Excessive sucking	1	Excessive sucking	1
Poor feeding	2	Poor feeding	1
Regurgitation	2	Regurgitation	1
Projectile vomiting	3	NA	NA
Stools		NA	NA
Loose	2	NA	NA
Watery	3	NA	NA

Abbreviation: FNAST, Finnegan Neonatal Abstinence Scoring Tool; NA, not applicable.

^a^ Cells marked with NA indicate that the original binary item was not retained in the simplified FNAST.

**Table 5.** Comparison of Cutoffs for Initiation of Pharmacologic Treatment Between the Original 21-Item and Simplified 8-Item FNAST Scores Among 424 Neonates

Original FNAST cutoff	Neonates, No. (%)^a^			
	Simplified FNAST cutoff			Total
	0-3	4	≥5	
0-7	87 (20.5)	13 (3.1)	2 (0.5)	102 (24.1)
8-11	47 (11.1)	58 (13.7)	23 (5.4)	128 (30.2)
≥12	25 (5.9)	41 (9.7)	128 (30.2)	194 (45.8)
Total	159 (37.5)	112 (26.4)	153 (36.1)	424 (100)

Abbreviation: FNAST, Finnegan Neonatal Abstinence Scoring Tool.

^a^ The weighted κ was 0.55 (95% CI, 0.48-0.61).

## Discussion

In this cohort study, a simplified, binary, 8-item FNAST scale was developed to discriminate between neonates who did and did not receive pharmacologic therapy based on traditional FNAST parameters (Table 4; eTable in the Supplement). The items were subsequently validated with an independent cohort of neonates with opioid exposure. The logistic model with the items on the simplified scale discriminated nearly as well as the model that incorporated the original FNAST items (AUC, 0.86 [95% CI, 0.82-0.89] vs 0.90 [95% CI, 0.87-0.94]) despite dichotomizing and eliminating many of the items. The components of the FNAST identified in this 8-item model performed well despite variation in the algorithm used to initiate pharmacologic therapy among the cohorts (ie, 2 consecutive FNAST scores of ≥8 or 1 of ≥12; 3 consecutive scores ≥8 or 2 of ≥12), and they did not lose predictive power in an independent validation sample that used a different algorithm (ie, the MNS) for assessing the need for pharmacologic therapy. The high AUC that we observed in the validation cohort is evidence of the transportability of the 8-item simplified scale, especially since the MNS scale differed from the FNAST by including some signs that were not in the FNAST and excluding others that were.

When the FNAST and simplified FNAST scores were categorized according to treatment cutoffs, the agreement was only moderate (weighted κ = 0.55; 95% CI, 0.48-0.61). There are 2 cutoffs for the original FNAST that were predetermined at 8 and 12 points, leaving little room to set the simplified FNAST thresholds to optimize agreement. In practice, the treatment decision is based on frequent assessments, not a single score. Because there is no criterion standard, there is currently no method for determining whether an algorithm based on the original FNAST or the 8-item version would yield better clinical outcomes.

The model did not select some signs because they occurred infrequently or almost always and hence were not useful in predicting pharmacologic treatment for NAS. Not every item that was associated with the outcome in univariate analyses made it into the final logistic regression model. Having a high odds ratio in a univariate analysis does not ensure retention in a multivariable model if an item is associated with other items and/or the confidence interval is wide. Signs that were absent from the final model may still be clinically important, although they do not have to be checked to make a treatment decision.

Each item on the original FNAST was weighted and based on clinical observations at the time the tool was developed. Discerning the degree of expression of several items on the original FNAST has proven difficult.^35^ Differences in the assessment of withdrawal severity have been associated with suboptimal short-term outcomes in neonates with NAS.^36^ In this study, we evaluated differences in the scoring of individual items of the FNAST among different cohorts (Table 3) and found significant differences in the frequency of some items. The most marked differences were noted with high-pitched crying and hyperactive Moro reflex, which were excluded from further analysis.

Several simplified Finnegan-based scoring tools have been developed.^27,30,32,33^ The 10-item simplified Finnegan Neonatal Abstinence Scoring System (sFNAST),^33^ published in 2017, included 7 of 8 items in the scale proposed in this study (ie, tremors, increased muscle tone, poor sleep, tachypnea, excessive sucking, poor feeding, and feeding intolerance). This overlap is notable considering the differences in method for deriving the scores. The sFNAST study focused on neonates who received treatment, used multiple assessments from each patient, selected items based on correlation with the original FNAST, and did not have enough sites to evaluate heterogeneity. The present study included large numbers of neonates with and without pharmacologic treatment, used the highest score on the day treatment was initiated (or day 3 of life if never treated), selected items to optimize discrimination, excluded items with marked differences among cohorts, and was validated using an independent cohort.

A 5-item MNS Short Form was developed based on the ability to discriminate between neonates receiving pharmacologic treatment and those who were not treated.^32^ The MNS Short Form shares 3 items with our scale (ie, tremors, increased muscle tone, and tachypnea). One item on the MNS Short Form (excessive irritability) is not on the original FNAST. Similar to our study, there was a single score per neonate and the goal was optimization of discrimination. The AUC of the 5-item MNS was 0.90, which was close to the AUC of 0.94 for the 19-item MNS. However, there was no independent validation sample. Furthermore, because data came from a single clinical trial, site-to-site homogeneity may have been more favorable than it would have been in a sample of unrelated sites.

Our proposed scale overlaps with the Eat, Sleep, and Console (ESC) assessment method.^37,38,39^ This method assesses if a neonate with opioid exposure can eat at least 1 ounce (or age appropriate volume) per feed or breastfeed well, sleep at least 1 hour, and be consoled within 10 minutes. While the ESC approach has gained acceptance at many sites in the United States since the initial quality improvement study introduced the approach in 2017, the generalizability and safety of the approach has yet to be demonstrated. Tachypnea, which is a key sign of central nervous system irritability and autonomic dysfunction, is not assessed with the ESC approach. However, it was retained in our simplified FNAST, in the sFNAST, and in the 5-item MNS.

### Limitations

There are several limitations in this study. First, data were retrospective for some of the cohorts and prospective for others. Next, most binary FNAST items had statistically significant differences among cohorts. The 2 items that were eliminated because of pronounced sample differences in percentage endorsement (ie, high-pitched crying and hyperactive Moro reflex) could perhaps be reintegrated in the future, if consistency in assessment can be achieved. Despite the statistically significant variability of some of the included items, the simplified score’s ability to discriminate neonates who were treated vs those who were untreated (as measured by the AUC) was very high, even in the validation cohort. Shorter tools have been published (ie, the 5-item MNS and the ESC), but we retained 8 items because of the trade-off between brevity and potential loss of generalizability. Further prospective studies that incorporate nursing and caregiver input are needed to establish clinical utility and determine the validity and reliability of this simplified tool in comparison with other tools and approaches.

## Conclusions

In this study, the 21-item FNAST was simplified to an 8-item scale that discriminated nearly as well as the original and was validated with an independent cohort. This shorter assessment tool could simplify clinical assessment by focusing on components that are relatively consistent across sites. It is important to prospectively validate this scale, which could be widely used and lead to the standardization of the clinical approach and management of neonates prenatally exposed to opioids.

## Notes

Supplement. **eFigure.** Flow Diagram for Patient Inclusion **eTable. **Simplified FNAST Evaluation Tool
