# Supplementary material for: Association of a Simplified Finnegan Neonatal Abstinence Scoring Tool With the Need for Pharmacologic Treatment for Neonatal Abstinence Syndrome
Source: JAMA Netw Open. 2020 Apr 8;3(4):e202275. doi: 10.1001/jamanetworkopen.2020.2275 (PMC7142377; doi:10.1001/jamanetworkopen.2020.2275)
Supplement: Supplement. — eFigure. Flow Diagram for Patient Inclusion eTable. Simplified FNAST Evaluation Tool [file jamanetwopen-3-e202275-s001.pdf]

Supplementary Online Content

Devlin LA, Breeze JL, Terrin N, et al. Association of a simplified Finnegan Neonatal Abstinence Scoring Tool with the need for pharmacologic treatment for neonatal abstinence syndrome. *JAMA Netw Open*. 2020;3(4):e202275. doi:10.1001/jamanetworkopen.2020.2275

eFigure. Flow Diagram for Patient Inclusion

eTable. Simplified FNAST Evaluation Tool

This supplementary material has been provided by the authors to give readers additional information about their work.

© 2020 Devlin LA et al. *JAMA Network Open.*

eFigure. Flow Diagram for Patient Inclusion

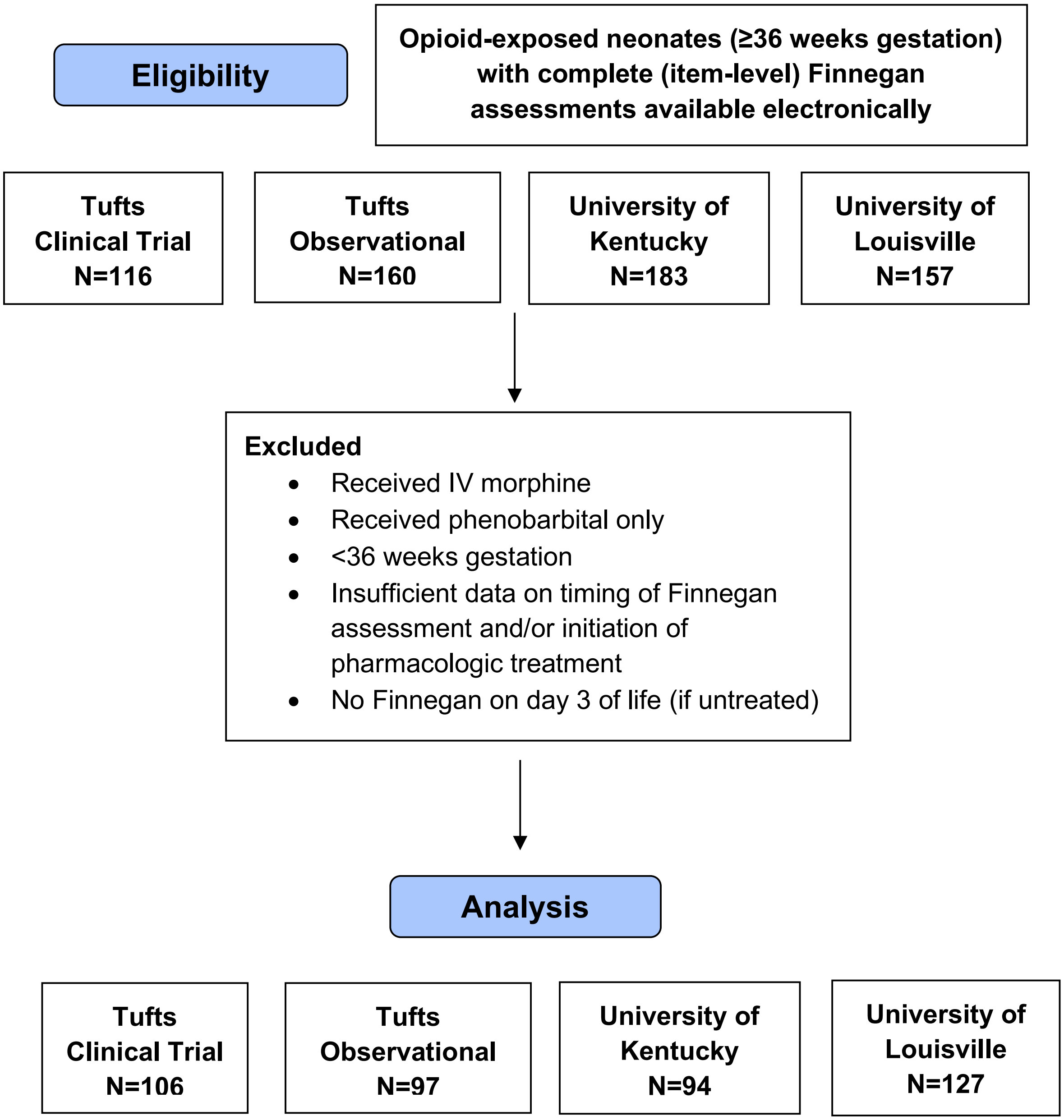

© 2020 Devlin LA et al. *JAMA Network Open.*

eTable. Simplified FNAST Evaluation Tool

**Item**		**Score**
**Sleeps <3 Hours After Feeding**	☐Present<br>☐Absent	1
**Any Tremors**	☐Present<br>☐Absent	1
**Increased Muscle Tone**	☐Present<br>☐Absent	1
**Fever >=37.2°C**	☐Present<br>☐Absent	1
**Respiratory Rate >60/min**	☐Present<br>☐Absent	1
**Excessive Sucking**	☐Present<br>☐Absent	1
**Poor Feeding**	☐Present<br>☐Absent	1
**Regurgitation**	☐Present<br>☐Absent	1
**During the assessment period:**<br>**Patient receives a score of 1 if an item is present or a score 0 if item is absent**<br>**Total Score is the sum of all items that are present**		**Total Score**

© 2020 Devlin LA et al. *JAMA Network Open.*
